# The Boston Medical and Surgical Journal’s Seventy-Fifth Anniversary

**Published:** 1903-04

**Authors:** 


### The Boston Medical and Surgical Journal's Seventy-Fifth Anniversary.

IN A modest editorial in its issue of February 19, 1903, our sterling and esteemed contemporary commemorates its seventy-fifth year! It merely announces the fact in a few plain words, that its first number appeared February 19, 1828, and that there has been a continuous weekly issue of the *Journal* since that time. It mentions the further fact that in another form its birth antedates 1828, because it was the product of the consolidation of the *New England Medical Journal*, begun in 1812, and the *Boston Medical Intelligencer*, established in 1823, and adds that the *Boston Medical Journal* was not only the first, but for many years the only weekly medical journal in this country. Reference then is made to some journalistic history, to an article by Dr. Billings on the Medical Journals of the United States, published in 1878, and to the impersonal editorial and business management of the *Journal*. And that is all!

The prolonged and honorable career of our Boston contemporary is the pride of every American medical editor, and it gives us great pleasure to offer our congratulations to the "dean" of medical journals, not only for the age it has attained, but for the course it has pursued during all its years,—a course at once honorable, conservative, progressive, scientific and distinguished. It is a record that may well stimulate the pride of all concerned in editing and publishing the *Journal*, and we cannot do more than to wish it continued prosperity on the lines it has marked out for itself. To every one connected with the conduct of the *Journal* we beg to say—"May you all live long and prosper."

---

A LITERATURE class for nurses has been instituted by Dr. Edwin Walker, of Evansville, Ind., at his sanitorium and training school. It has been in successful operation for some time and has attracted the attention of physicians and nurses far and wide. In the *Trained Nurse and Hospital Review*, Miss Rosalie Isabel Stewart presents the scheme in an interesting manner. It was she who, in association with a high school professor of literature, laid out a three-year course of study in literature for Dr. Walker's nurses. It is a most beneficial, instructive, and even amusing way in which nurses can be entertained when off duty, and deserves the attention of officers of training schools.

---

SEVERAL newspapers in this city have of late flaunted in the faces of the people advertisements so offensive in character that the grand jury of the county court lately felt constrained to administer the following censure:

The grand jury of the county of Erie for February, 1903, believing that the newspapers of Buffalo are and ought to be powerful factors in the improvement of existing conditions in matters affecting our moral and municipal life, therefore, this grand jury in performance of its public duty, does hereby express its emphatic disapproval of the course of some of our reputable newspapers, in allowing the use of their columns for advertising transparent fakes and frauds for deceiving the public. The grand jury believes that in so doing these newspapers are lending their aid to schemes for fleecing the credulous and unwary instead of protecting the interests of the public by exposing such frauds or by refusing to publish such advertisements.

CHARLES N. BRAYTON, Foreman.

GEORGE R. HOWARD, Secretary.

Possibly, in case this admonition is inadequate to check the evil, another grand jury may take a step further in the premises.

---

THE New York Central & Hudson River Railroad, it is announced, will employ sixty surgeons along its route, between New York and Buffalo, to be called upon in case of the injury of passengers or employees. These surgeons are located at conveniently accessible distances. In case of accident the medical officer having charge of the section of road on which it occurs is to be notified at once and brought to the scene with all speed to render prompt aid to the injured.

These physicians will also be called by telegraph in cases of persons taken ill on trains and will minister to the physical ills of employees of the company. Specialists have also been employed to render service in cases in which their skill may be needful to cure the results of injuries or where a difficult operation may be necessary.

---

AFTER hearing General Leonard Wood, who delivered an after-luncheon address to the Liberal Club, March 7, 1903, it would not be easy to deny the thoroughness with which the sanitary work has been done in Cuba. Let us hear no more scepticism regarding the prophylaxis of vaccination when General Wood tells us that a regiment of about 800 men was vaccinated and revaccinated and then was detailed to care for 3,000 smallpox patients, but not one soldier caught the disease! During the last year of the occupancy the American army lost but fourteen men, eight of whom were drowned or otherwise accidentally killed. So much for intelligent sanitation in a country where there had been three hundred years of neglect.

---

IT IS reported that a child three years old died recently from eating pills, left by a distributor of samples on the steps of a residence on Grant Street in this city. Another child two years older, it is reported, was made ill from the same cause. Is it not possible to put a stop to the annoying and, as shown by the circumstances, dangerous practice of house-to-house distribution of samples?

---

The secretary of the board of medical examiners, representing the Medical Society of the State of New York, reports as follows regarding the January, 1903, state medical licensing examinations: total number of candidates, 103; successful, 75 or 72 8-10 per cent.; unsuccessful, 28 or 27 1-10 per cent. Of this number 8 had previously passed in the medical primary branches. There was but 1 "honor" licentiate. Fifty-one candidates appeared for certificate of proficiency in the medical primary branches (anatomy, physiology, hygiene and chemistry). 47 or 92 1-10 per cent. of these were successful.

